# Depletion of CD4 and CD8 Positive T Cells Impairs Venous Thrombus Resolution in Mice

**DOI:** 10.3390/ijms21051650

**Published:** 2020-02-28

**Authors:** Subhradip Mukhopadhyay, Joel Gabre, Christine Chabasse, Jonathan S. Bromberg, Toni M. Antalis, Rajabrata Sarkar

**Affiliations:** 1 Center for Vascular and Inflammatory Diseases, University of Maryland School of Medicine, Baltimore, MD 21201, USA; jgabre@gmail.com (J.G.); c_chabasse@yahoo.fr (C.C.); jbromberg@som.umaryland.edu (J.S.B.); tantalis@som.umaryland.edu (T.M.A.); rsarkar@som.umaryland.edu (R.S.); 2 Department of Surgery, University of Maryland School of Medicine, Baltimore, MD 21201, USA; 3 Department of Physiology, University of Maryland School of Medicine, Baltimore, MD 21201, USA; 4 Veterans Affairs Health Care System, Baltimore, MD 21201, USA

**Keywords:** peripheral vascular disease, T cells, CD4, CD8, thrombus resolution, matrix metalloproteinase, macrophage

## Abstract

Resolution of deep venous thrombosis involves coordinated inflammatory processes. T cells regulate inflammation in vivo and modulate vascular remodeling in other settings, but their role in venous thrombus resolution remains undefined. To determine the role of T cells in venous thrombus resolution in vivo, stasis induced thrombi were created by vena cava ligation in outbred CD-1 mice. CD4 and CD8 positive T cells, as determined by flow cytometry, were present in thrombi both during thrombus formation and resolution. Depletion of the CD4 and CD8 positive T cells by antibody treatment selectively impaired thrombus resolution compared to animals treated with isotype control antibodies, without an effect on venous thrombus formation. Quantitation of intra-thrombus macrophage numbers, fibrinolytic marker expression, and gelatinolytic activity by zymography revealed that T cell depletion decreased the number of macrophages, reduced the expression of fibrinolytic marker urokinase plasminogen activator (uPA), and decreased the activity of matrix metalloprotinease-9 (MMP-9). These data implicate CD4 and CD8 positive T cells in functionally contributing to venous thrombus resolution, thus representing a potential therapeutic target, but also underscoring potential risks involved in T cell depletion used clinically for solid organ and hematopoietic transplantation procedures.

## 1. Introduction

Deep vein thrombosis (DVT) affects approximately 2,000,000 people in the US each year [1,2]. It significantly affects quality of life in patients and is a major burden to health care costs [3,4]. Individuals affected with DVT are at a high risk of potentially fatal pulmonary embolism (PE), as well as long-term complications of DVT including post-thrombotic syndrome (PTS) and chronic thromboembolic pulmonary hypertension (CTEPH) [5,6,7,8,9]. Current treatment for DVT involves anticoagulation, which effectively prevents propagation of the existing thrombus as well as new thrombus formation but fails to resolve already formed thrombus [10,11,12]. Despite the use of anticoagulants, approximately 25% to 50% of DVT patients develop PTS [13,14], whereas about 5% of patients suffering from an unresolved PE develop CTEPH as a late complication [15]. The lack of specific therapeutic options for these debilitating complications of DVT thus warrants a better cellular and molecular understanding of the process of venous thrombus formation and resolution. 

Current understanding of the molecular mechanisms involved in venous thrombus formation and resolution is largely derived from the use of rodent models of caval thrombus resolution, where the inferior vena cava of the animal is either completely or partially ligated to induce thrombosis [16,17,18,19,20]. Platelets along with the early thrombus infiltrating cells, such as neutrophils and monocytes, play a major role in the thrombus formation [21,22,23,24], whereas monocytes and macrophages play a major role in the resolution of the formed thrombus [25,26,27,28,29,30]. Thrombus resolution is a phase that is dominated by fibrinolysis and inflammatory vascular remodeling processes involving clot retraction, tissue clearing, and fibrotic changes [31,32,33]. Thus, venous thrombus resolution, in a sense, mimics wound healing. Numerous studies of venous thrombus resolution have documented the early influx of neutrophils and later migration of monocytes and macrophages into a resolving thrombus, and these inflammatory cells are a major source of cytokines and proteases that are involved in the process of venous thrombus resolution [34,35,36,37].

On the other hand, the role of immune cells, specifically T cells, in the process of venous thrombus resolution is less clear. Although early studies identified the presence of lymphocytes in the venous thrombus [38], their functional role in the process of venous thrombus resolution has only recently been examined. Using a mouse stasis induced venous thrombosis model, Frey et al. showed that B cell depletion in animals resulted in impaired thrombus resolution [39]. Luther et al. [40] recently investigated the role of effector memory T cells (T_EM_) in a mouse stenosis induced venous thrombosis model. Antibody mediated ablation of CD4^+^ and CD8^+^ T cells and recovery for 7 days was used to specifically deplete the T_EM_ population only. This treatment was found to accelerate venous thrombus resolution, however the overall contribution of CD4^+^ and CD8^+^ T cells to this process remains unknown. 

CD4^+^ and CD8^+^ T cells play a major role in the initiation and perpetuation of inflammatory cascades that involve crosstalk with other immune cells, including modulation of macrophage inflammatory status via secretion of cytokines [41,42,43,44]. Data from mouse models of atherosclerosis suggest a role for various T cell subsets in the development and progression of atherosclerosis. Antibody mediated depletion of CD4^+^ T cells has been shown to inhibit progression of atherosclerotic disease in mice [45]. T cells have been shown to confer protection against pulmonary angioproliferation, as evidenced by pulmonary arteriole occlusion with proliferating endothelial cells, mast cells, B cells, and macrophages in athymic rat lung lacking any T cells [46]. In a rat balloon catheter induced carotid artery injury model, athymic nu/nu rat lacking T cells had increased neointima formation [47], whereas adoptive transfer of CD4 and CD8 cells to Rag-1 mice after carotid artery injury was associated with reduced neointima formation [48]. 

To investigate the contribution of CD4^+^ and CD8^+^ T cells in the processes of venous thrombus formation and resolution, we actively depleted CD4^+^ and CD8^+^ T cells prior to venous thrombus formation and during thrombus resolution. The results showed that depletion of CD4^+^ and CD8^+^ T cells impairs thrombus resolution but not formation. T cell depletion lead to fewer infiltrating macrophages in the resolving thrombus, and this was also associated with reduced uPA and MMP-9 in the T cell depleted thrombi. Since depletion of T cells is used clinically in patients during solid organ transplant and hematopoietic stem cell transplantation procedures [49,50], our finding underscores the risks associated with the use of T cell depletion and its effect on venous thromboembolisms.

## 2. Results

### 2.1. T Cells Are Present in Both Early and Late Stages of Venous Thrombosis

**Figure 1:**
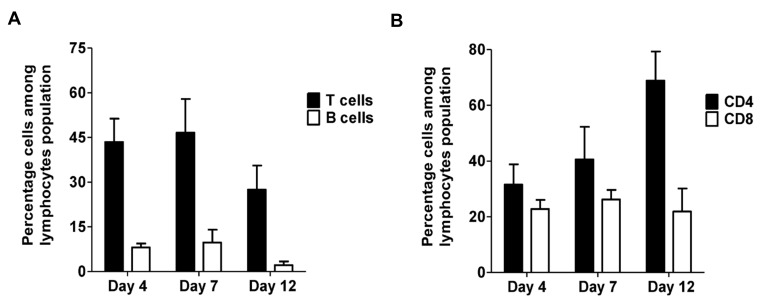
T cells and B cells are present in venous thrombus. (**A**) Quantitative analysis of the percentage of B cells (B220) and T cells (CD3) among the lymphocyte populations in the thrombus lysate. All values represent the mean ± SEM. (*n* = 2–4 per time point). (**B**) Quantitative analysis of percentage of CD4^+^ and CD8^+^ cells among the T lymphocyte population in the thrombus lysate. All values represent the mean ± SEM. (*n* = 2–4 per time point).

In our animal model of deep vein thrombosis, a thrombus formed after vena cava ligation reaches it maximum size at 4 days post-ligation and then gradually decreases in size via natural thrombus resolution. Thrombus weight at day 4 after the vena cava ligation is thus considered a measure of thrombus formation, whereas thrombus weight at 12 days is used as a measure of thrombus resolution in this model. We first sought to establish if T cells were present in venous thrombi. To test this, single cell suspensions of thrombus lysates obtained at different time points after vena cava ligation were assessed by flow cytometry. As shown in Figure 1A, lymphocytes were present in the thrombus throughout thrombus formation and resolution. The lymphocyte cell population contained both B cells (B220 positive) and T cells (CD3 positive). It is interesting to note that similar to peripheral blood, the absolute number of T cells was far greater than the B cells (Figure 1A) in the resolving thrombus. Both B cell and T cell populations peaked at day 7 (Figure 1A). We then further characterized the T cell population present in the resolving thrombus by CD4 and CD8 subset phenotype. Both CD4^+^ and CD8^+^ T cells were present in the thrombus (Figure 1B) and the relative percentages of CD4^+^ and CD8^+^ T cells approximated the normal ratios of these cells present in the peripheral blood [51]. These findings suggest that B cells and T cells in a resolving thrombus might accumulate through passive mechanisms. 

### 2.2. T Cell Depletion Impairs Venous Thrombus Resolution

**Figure 2:**
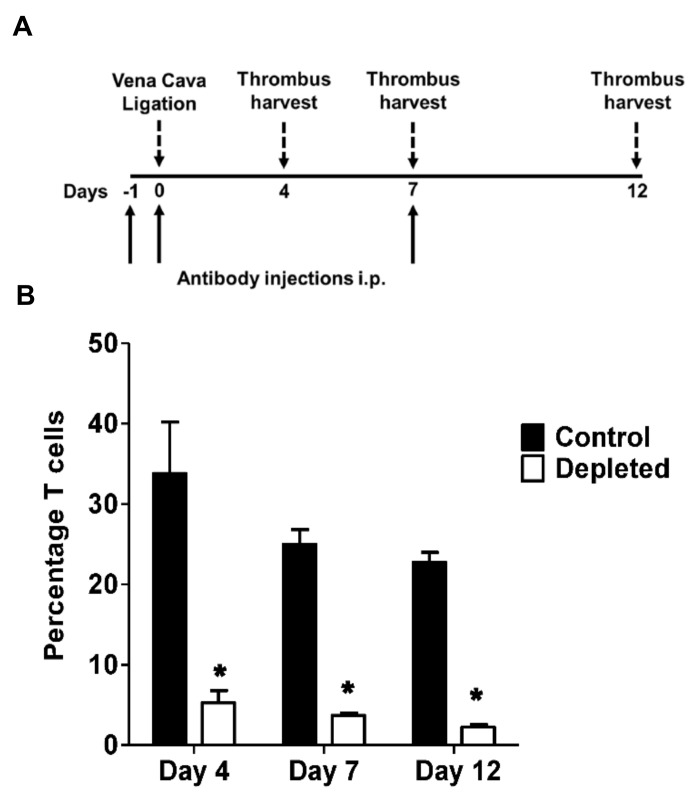
Depletion of T cells in animals impairs venous thrombus resolution. (**A**) Schematic representation of the experimental plan. Solid arrows indicate time-points (days) at which intraperitoneal injection of the antibodies were performed. (**B**) Quantitative analysis of CD3 positive T cells by flow cytometry of spleen lysates from control and T cell depleted animals at various post-surgery days. All values represent the mean ± SEM. (*n* = 3–4 per time point). *p < 0.05, control versus T-cell depleted.

We next investigated the functional role of T cells in venous thrombosis by comparing venous thrombus formation induced by vena cava ligation between animals treated with both anti-CD4 and anti-CD8 antibodies (depleted) or isotype matched antibodies (control) (Figure 2A). Flow cytometric analysis confirmed that anti-CD4 and anti-CD8 antibody treatment effectively reduced the total T cell numbers in the spleens of the treated animals compared to the control animals (Figure 2B). 

**Figure 3:**
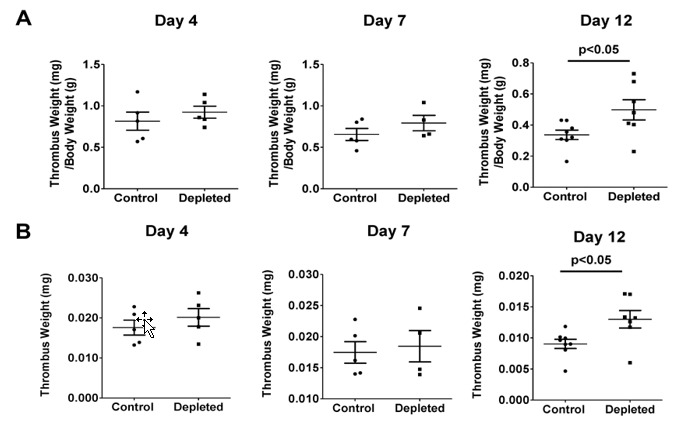
Depletion of T cells impairs venous thrombus resolution. (**A**) Thrombus weight normalized to mouse body weight over time after vena cava ligation in T cell depleted mice compared to control mice (Day 4 *n* = 5, Day 7 *n* = 4–5, Day 12 *n* = 7–8). (**B**) Thrombus weight over time after vena cava ligation in T cell depleted mice compared to control mice (Day 4 *n* = 5, Day 7 *n* = 4–5, Day 12 *n* = 7–8).

Both anti–T cell and control antibody treated animals developed thrombi of comparable size at both day 4 and day 7 after the vena cava ligation (Figure 3A), indicating that the depletion of T cells did not modulate venous thrombus formation or early thrombus resolution. In contrast to the thrombi harvested at earlier time points, T cell depleted animals had significantly larger thrombi at day 12 after vena cava ligation, suggesting that depletion significantly impairs processes during late resolution (Figure 3A). The increased thrombus weight at day 12 remained significant when thrombus weight alone was analyzed (Figure 3B).

### 2.3. T Cell Depleted Thrombi Have Decreased Macrophage Infiltration

**Figure 4:**
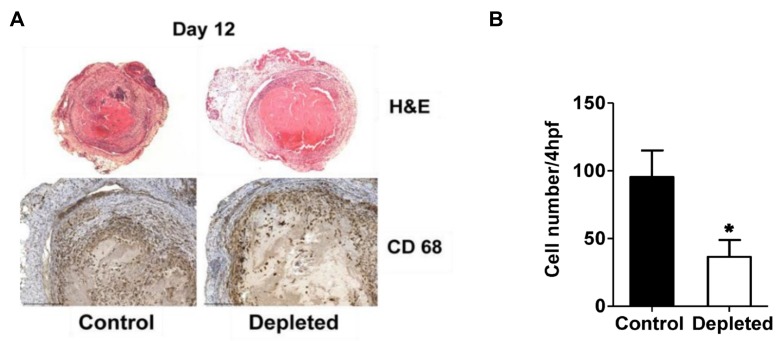
Depletion of T cells reduces intra-thrombotic macrophage numbers. (**A**) Histochemical analysis by H&E staining of venous thrombi sections from control and T cell depleted mice at 12 days after vena cava ligation (upper panel) and immunohistochemical analysis of intra-thrombotic macrophage accumulation using anti-CD68 antibodies in venous thrombi sections from control and T cell depleted mice at 12 days after vena cava ligation (lower panel). Original magnification, ×100 upper panel, ×200 lower panel. Representative results from 4–5 independent animals are shown. (**B**) Quantification of the numbers of CD68 positive cells (macrophages). All values represent the mean ± SEM (*n* = 4–5). *p < 0.05, control versus T-cell depleted.

H&E staining of histological sections from the control and T cell depleted thrombi at post-surgery day 12 showed no morphological differences between the two groups of thrombi (Figure 4A, upper panel). Macrophages are recognized to play a major role in venous thrombus resolution and are the predominant cell type present in the resolving thrombi in the late phase [27,28,29]. To determine whether the depletion of T cells had an effect on macrophages during the late stage of thrombus resolution, we quantitated the macrophage numbers that had infiltrated within the venous thrombi at Day 12. Thrombi sections from T cell depleted animals showed reduced staining for CD68 positive macrophages at day 12 after the vena cava ligation (Figure 4A, lower panel), and quantitation of thrombi sections for CD68 positive macrophage numbers confirmed T cell depleted animals had significantly reduced infiltrating macrophage numbers in the thrombi (Figure 4B). 

### 2.4. T Cell Depletion Decreases Fibrinolytic Activity in the Resolving Thrombus

**Figure 5:**
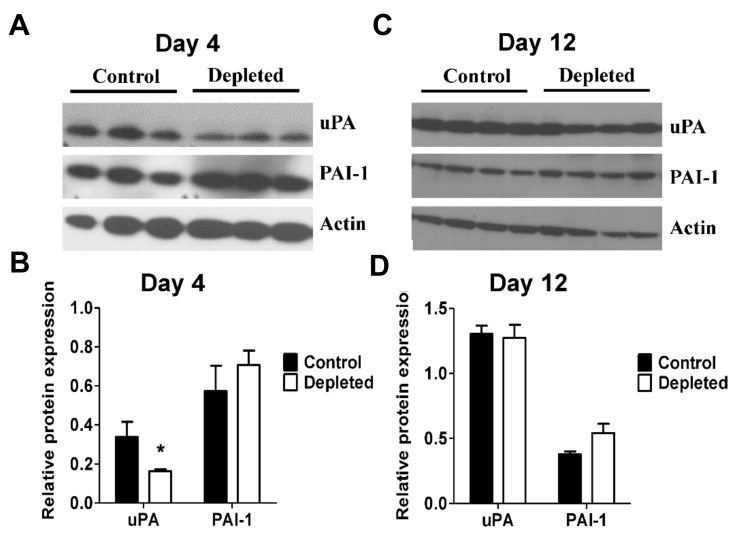
T cell depleted thrombi has reduced fibrinolytic activity. (**A**) and (**C**) Immunoblot analysis of intra-thrombotic fibrinolytic markers uPA and PAI-1 at days 4 and 12 after vena cava ligation in venous thrombus samples from control and T cell depleted mice (Day 4 *n* = 3, Day 12 *n* = 4). (**B**) and (**D**) Quantitative analysis of the expression of uPA and PAI-1. All values represent the mean ± SEM. *p < 0.05, control versus T cell depleted.

Degradation of fibrin by plasmin after the initial thrombus formation is a major mechanism that results in reduction of the venous thrombus burden. Macrophages play a major role in the modulation of the fibrinolytic activities by secretion of the plasminogen activator uPA and its inhibitor plasminogen activator inhibitor-1 (PAI-1) [52,53,54]. We tested whether T cell depletion modulated fibrinolytic activity in the venous thrombi. Immunoblot analyses showed significantly reduced levels of uPA protein expression in the thrombus lysates from T cell depleted animals at post-ligation day 4 (Figure 5A,B), whereas uPA expression was unaffected at post-ligation day 12 (Figure 5C,D). Expression of PAI-1 was not significantly changed by T cell depletion at both days 4 and 12 after vena cava ligation (Figure 5A–D). These data suggest modulation of early fibrinolytic activity in the resolving thrombus by T cells. 

### 2.5. T Cell Depleted Thrombi Have Decreased MMP-9 Activity 

**Figure 6:**
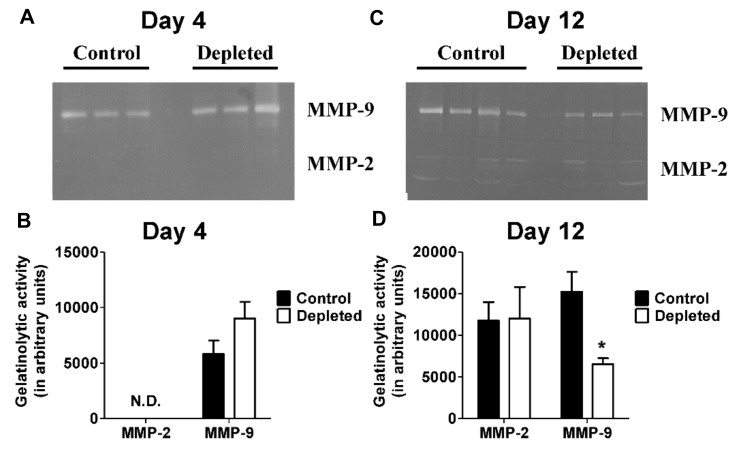
Depletion of T cells reduces intra-thrombotic gelatinolytic activity. (**A**) and (**C**) Gelatin zymogram gel images of intra-thrombotic MMP-2 and MMP-9 activities in venous thrombus samples from control and T cell depleted mice (Day 4 *n* = 3 control and 3 depleted, Day 12 *n* = 4 control and 3 depleted). (**B**) and (**D**) Quantitative analysis of gel images. N.D. = not detected. All values represent the mean ± SEM. *p < 0.05, control versus T cell depleted.

Thrombus resolution involves matrix remodeling and previous studies have implicated both MMP-2 and MMP-9 in mediating collagen remodeling during venous thrombus resolution [34,55]. Measurement of MMP-2 and MMP-9 activities in the resolving thrombi by gelatin zymography at post ligation day 4 showed no detectable MMP-2 activity in T cell depleted and control thrombus lysates, and no differences in MMP-9 activity (Figure 6A,B). On the other hand, MMP9 activity was significantly decreased in T cell depleted animals at post ligation day 12 (Figure 6C,D). There were no significant differences in MMP-2 activity at post-ligation day 12 between the groups (Figure 6C,D). These data suggest that depletion of T cells leads to decrease of MMP-9 activity in the resolving thrombus.

## 3. Discussion

Here, we show that CD4^+^ and CD8^+^ T cells are present in venous thrombi throughout thrombus formation and resolution and are required for thrombus resolution. Depletion of CD4^+^ and CD8^+^ T cells prior to thrombus formation had no effect on the acute phase of DVT but reduced macrophage infiltration during the resolution process. 

In addition to their roles in adaptive immunity, T cells are major modulators of inflammation [56,57]. The CD4^+^ subset of T cells are the regulators of the adaptive immune response [58], whereas the CD8^+^ subset, also known as cytotoxic T cells (Tc cells) are involved in the defense mechanism against various intracellular pathogens as well as cancer cells [59]. T cells and T cell subsets are well recognized to play a major role in the maintenance and propagation of immune and inflammatory processes in vivo and in recent years, their involvement in the pathophysiology of vascular diseases such as DVT has been recognized. DVT involves infiltration of the thrombus by leukocytes during the early stages. Neutrophils are the first to infiltrate a resolving thrombus and are then followed by monocytes and macrophages [21]. The initial thrombus is rich in fibrin and as the thrombus matures, it undergoes fibrinolysis mediated by factors largely secreted by thrombus associated macrophages. Maturation of the thrombus is also characterized by collagen deposition and remodeling [60]. Thrombus associated leukocytes secrete various cytokines and regulatory factors that modulate the processes that are required for venous thrombus resolution [36]. Using a mouse stenosis model of DVT, Luther et al. [40] showed that T_EM_ in the thrombus vein wall undergo an antigen-independent activation and produce IFN-gamma. Specific depletion of the T_EM_ subset resulted in accelerated venous thrombus resolution. These results are in agreement with the previous finding that mice lacking the IFN-gamma gene have accelerated thrombus resolution [34]. All these data suggest that the change in the inflammatory milieu in the resolving venous thrombus directly influences the outcome of the resolution process. 

Our data showing that impairment of thrombus resolution after depletion of CD4^+^ and CD8^+^ T cells is in contrast with Luther et al. [40] who observed accelerated thrombus resolution after T cell depletion. It is important to point out that Luther et al. used antibodies to deplete the T cell population and the mice were subsequently allowed to recover to refill their T cell compartment with newly generated naïve T cells, so the animals were specifically T_EM_ cells depleted. The different outcomes suggest that various subsets of T cells modulate the process of thrombus resolution in divergent manners. In our study, antibody mediated depletion of CD4^+^ and CD8^+^ T cells was associated with lower numbers of infiltrating macrophages in the T cell depleted thrombi. Cytokines secreted by activated T cells are known to influence the recruitment, activation, and maturation of macrophages [61] and, conversely, interaction of activated monocytes and macrophages with T cells significantly modulates T cell activities [43]. Our finding that there are reduced numbers of macrophages in the T cell depleted thrombi suggests that T cells might play a major role in modulating the homing and/or maturation of macrophages in a resolving venous thrombus, leading to impaired venous thrombus resolution. Macrophages are a major source of fibrinolytic and collagenolytic pathways through uPA [52,53,54] and MMP-9 [34,55], respectively, during thrombus resolution. Our data showing that decreased macrophage content is associated with reduced uPA and MMP-9 levels in the T cell depleted thrombi is congruent with these previous findings.

Our observations are significant given the fact that depletion of host T lymphocytes is a common clinical practice during solid organ transplant as well as allogeneic stem cell transplantation procedures [49,50]. It is currently not known how T lymphocyte depletion affects the resolution of preexisting venous thrombus in patients. Furthermore, the recent development and use of immune checkpoint inhibitors, such as anti PD-1 and anti CLTA4, for cancer immunotherapy [62] also raise questions regarding their effect on thrombus resolution, as these inhibitors exert their effects by modulating T cell activity. Further research involving preclinical animal models of venous thrombosis and immune checkpoint inhibitors is needed to elucidate these molecular interactions. 

## 4. Materials and Methods

### 4.1. Murine Stasis Induced Venous Thrombosis 

Mouse stasis induced venous thrombosis by vena cava ligation is a well-established model and has been previously described [30,33,34,36,37]. Briefly, 10 to 14 weeks old CD-1 outbred mice (Charles River Laboratories, Wilmington, MA, USA) underwent general anesthesia using inhaled 2%–3% isoflurane and a laparotomy was performed to expose the retroperitoneal area. The inferior vena cava (IVC) was then separated from the abdominal aorta and ligated with a 6-0 silk suture immediately distal to the renal branches. All side branches of the IVC were cauterized, producing a stasis induced venous thrombus. At different time points after the ligation of the vena cava, thrombus resolution was monitored by harvesting the IVC containing the thrombus between the renal veins and the iliac bifurcation. Thrombi were weighed and stored at −80 °C for protein and mRNA analysis or placed in formalin followed by 70% ethanol for histological analysis. All procedures were performed as approved by the Institutional Animal Care and Use Committee of University of Maryland Baltimore. 

### 4.2. T Cell Depletion 

For T cell depletion experiments, animals were treated with anti-CD4 (clone GK1.5) and anti-CD8 (clone 2.43) monoclonal antibodies (1 mg each) or 2 mg of isotype matched control antibody (clone LTF-2) (Bio X Cell, West Lebanon, NH, USA) via intraperitoneal injections on the days −1, 0, and +7 relative to the vena cava ligation. Depletion of greater than 97% CD4^+^ and CD8^+^ T cells was confirmed by flow cytometric analysis of T cell populations in the spleen as described in the flow cytometry section. Thrombi harvested from separate cohorts of age and sex matched animals undergoing the same treatment protocol were used to generate thrombus weight data/histological analyses and molecular studies.

### 4.3. Venous Thrombus Digestion

Thrombi were harvested on days 4, 7, and 12 after the vena cava ligation and were carefully cut into small segments using sterile forceps. Segments of the thrombi were processed into single cell suspensions using collagenase type II (Worthington Biochemical Corporation, Lakewood, NJ, USA) and mouse tPA (Molecular Innovations Inc., Novi, MI, USA). The treated suspension was filtered through a 40-micrometer cell strainer (BD Biosciences, San Jose, CA, USA) and centrifuged at 300× *g* to pellet the cells. Pelleted cells were subsequently resuspended in red cell lysis buffer (Sigma, St. Louis, MO, USA) for 3 min. Cells were then pelleted by centrifugation as above and resuspended in phosphate buffered saline and adjusted to a concentration of 1 × 10^6^ cells/mL and stained for one hour with appropriate antibodies prior to flow cytometric analysis. 

### 4.4. Flow Cytometric Analyses of Thrombus and Spleen

For T and B cell analyses, cells were stained with anti-CD3 FITC (clone 2C11), anti-B220 APC (clone RA3-6B2), anti-CD4-FITC (R&D Systems, Minneapolis, MN, USA), and anti-CD8 PE (clone Ly2) (eBiosciences, Waltham. MA, USA) and analyzed on a BD LSRFortessa (Becton Dickenson, San Jose, CA, USA). Forward and side scatter discriminated lymphocytes from other cells and debris. To ensure that the CD4 and CD8 antibody treatment resulted in depletion of CD4^+^ and CD8^+^ T cells and was not due to an artifact arising from down modulation or steric hindrance of the corresponding epitopes, we determined the global T cell numbers (using anti CD3 antibody) as well as the CD4^+^ and CD8^+^ T cell numbers in single cell suspensions from spleens collected at each time point of the thrombus harvest. Depletion of T cells was routinely found to be >97%.

### 4.5. Immunohistochemistry

Paraffin-embedded thrombus sections (5 μm) from animals harvested at day 12 underwent standard histochemical staining for H&E (Sigma) as well as immunostaining for macrophages using anti CD68 antibody (Abcam, Cambridge, MA, USA) following standard procedures.

### 4.6. Immunoblotting

Thrombi were homogenized in tissue protein extraction reagent (T-PER, Thermo Fisher Scientific, Waltham, MA, USA) and incubated at room temperature for 5 min. Then, 20 µg extracted proteins were separated by 12% SDS–polyacrylamide gel electrophoresis (PAGE), transferred onto PVDF membranes, and probed with the following primary antibodies: anti-beta-actin (Santa Cruz Biotechnology, Dallas, TX, USA), uPA (Pierce, Waltham, MA, USA), PAI-1 (American Diagnostica, Stamford, CT, USA). Immuno-reactive bands were visualized using an ECL chemiluminescence detection kit (Amersham, Piscataway, NJ, USA) and exposed by autoradiography. Quantification was performed by densitometry using ImageJ software and normalized with respect to beta-actin levels in the same sample. 

### 4.7. Gelatin Gel Zymography 

MMP-2 and MMP-9 activity was analyzed by gelatin gel zymography according to the manufacturer’s instruction (Thermo Fisher Scientific, Waltham, MA, USA). Briefly, equal amounts of proteins were mixed with an equal volume of 2X Tris Glycine SDS Sample buffer loaded onto the gel and separated by electrophoresis. Gels were washed for 30 min at room temperature in Zymogram Renaturation Buffer and incubated overnight in Zymogram Development Buffer at 37 °C. The gels were stained with 0.1% Coomassie Brilliant Blue R-2500 and destained in 5% methanol and 7% acetic acid. Gelatinolytic activity appeared as a clear band on a blue background. MMP-2 and MMP-9 activities were determined using ImageJ software and expressed as relative densities normalized to the total protein.

### 4.8. Statistical Analysis

All statistical analyses were performed using Student’s unpaired *t*-test. Differences were considered significant at *p* ≤ 0.05. All data are reported as mean ± standard error.

## Abbreviations

CTEPH	Chronic thromboembolic pulmonary hypertension
DVT	Deep vein thrombosis
IVC	Inferior vena cava
MMP	Matrix metalloproteinase
PAI-1	Plasminogen activator inhibitor type I
PE	Pulmonary embolism
PTS	Post-thrombotic syndrome
T_EM_	Effector memory T cells
tPA	Tissue-type plasminogen activator
uPA	Urokinase-type plasminogen activator
